# Multi-organ multi-omic and gut microbiome markers of fat and sucrose dietary oversupply in cardiometabolic disease

**DOI:** 10.1016/j.isci.2025.111887

**Published:** 2025-03-01

**Authors:** Ren Ping Liu, Alistair Senior, Zhen Bao, Yen Chin Koay, Andrew Holmes, John F. O’Sullivan

**Affiliations:** 1 Cardiometabolic Medicine Group, The University of Sydney, Sydney, NSW, Australia; 2 School of Medical Sciences, Faculty of Medicine and Health, The University of Sydney, Sydney, NSW, Australia; 3 Charles Perkins Centre, The University of Sydney, Sydney, NSW, Australia; 4 School of Life and Environmental Sciences, The University of Sydney, Sydney, NSW, Australia; 5 Sydney Centre for Precision Data Science, The University of Sydney, Sydney, NSW, Australia; 6 Department of Cardiology, Royal Prince Alfred Hospital, Camperdown, NSW, Australia; 7 Faculty of Medicine, Technische Universität Dresden, Dresden, Germany

**Keywords:** Diet, Microbiome

## Abstract

Cardiometabolic disease is the greatest challenge facing global health. Increasingly, evidence suggests that Western diet comprising an over-supply of energy from fat and sucrose leads to obesity, insulin resistance, hypertension, and cardiovascular disease. Traditional preclinical animal studies of cardiometabolic disease often adopt a reductionist approach, focusing on individual components. To overcome this, we comprehensively assessed cardiometabolic phenotypes— anthropometric, physiological, and metabolic— along with the molecular changes consequent upon fat or sucrose dietary oversupply, or both in male C57BL/6J mice. Molecular assessment included measurement of the gut microbiome and several metabolite pools including plasma, heart, liver, and gut contents (cecal and fecal). In these mice, we identified key changes across phenotypes, metabolites, microbiota, and their interrelationship, and synthesized all the data into four distinct phenogroups that explain the variance across cardiometabolic parameters. These phenogroups provide insight into inter-organ regulation of Western diet-dependent cardiometabolic phenotypes, highlighting important avenues for further study.

## Highlights

• Dietary fat oversupply caused spillover of methylated NAD products
• Fecal levels of SCFAs changed most on high-fat containing diets
• Thiamine was elevated in both HSD and HFD in plasma, heart, and liver

## Graphical abstract

**Figure:**
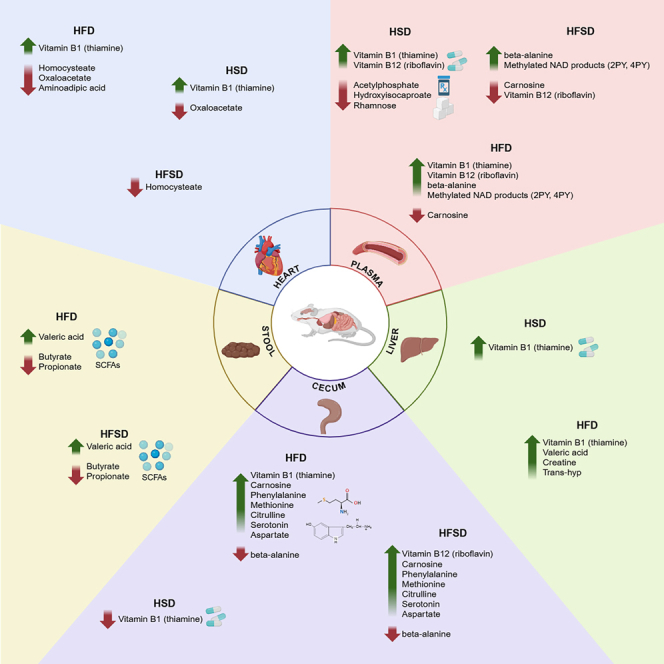

## Introduction

It is now recognized that cardiometabolic health is shaped by the intricate interplay between the dimensions of diet, the gut microbiome, and physiology, each of which is also complex in its own right.^1^ Dietary factors have been consistently associated with modulation of cardiometabolic risk factors including obesity, insulin resistance, dyslipidemia, and hypertension.^1^^,^^2^^,^^3^^,^^4^ High saturated fat diets, in particular, contribute to increased adiposity and ectopic fat deposition, exacerbating systemic inflammation and oxidative stress.^3^^,^^5^^,^^6^ Similarly, high sucrose diets, even in the setting of low and medium dietary fat content, are obesogenic and metabolically adverse.^7^

Diet also affects gut microbial ecology in ways that may potentiate effects on phenotype.^8^ The gut microbiome plays a pivotal role in modulating host physiology^9^^,^^10^^,^^11^^,^^12^ and metabolic homeostasis^13^^,^^14^ while also contributing to various aspects of energy regulation^10^^,^^13^^,^^15^ and immune function.^16^ The causal contributions of gut microbes to these outcomes are complex and underpinned by both direct and indirect mechanisms. The net result is the fostering of a dysbiotic gut environment conducive to metabolic derangements and chronic low-grade inflammation which are key drivers of cardiometabolic disorders.^17^^,^^18^

With recognition of this complexity, the application of multi-omic platforms has emerged as a pivotal avenue for unraveling the intricacies of dietary influences on cardiometabolic health dynamics.^19^ This present study aimed to assess the effect of three experimental diets termed High Fat (HFD), High Sucrose (HSD), and High Fat+High Sucrose (HFSD) diets on cardiometabolic health in mice. These diets introduced defined nutrient imbalances with respect to the reference standard chow AIN93G: Fat profile and amount manipulated by introduction of lard; protein content by manipulating casein; carbohydrate profile and amount by adjusting relative amounts of cellulose, starch, and sucrose (Table S1). We employed careful anatomical, physiologic, metabolic, cardiac, and multi-omic phenotyping to assess the impact on microbiome and metabolite pools across multiple body sites to expose the signatures of cardiometabolic risk and protection in response to the different diets.

## Results

### Physiological effects induced by nutrient-imbalanced diets

**Figure 1:**
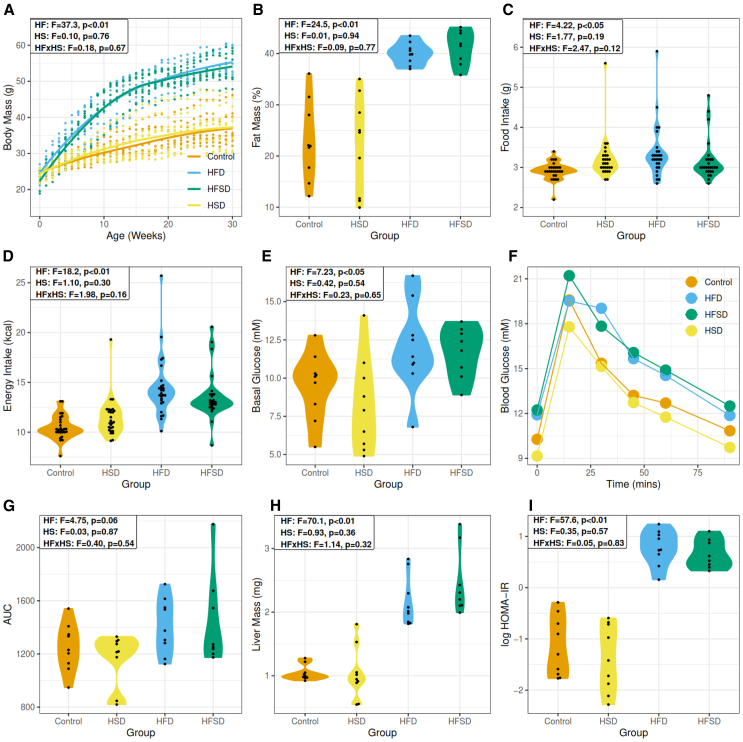
Diets imbalanced through inclusion of high saturated fat, led to systemic physiological changes in male C57BL/6J mice (A) Body mass in grams assessed each week over the 30-week study. (B) Fat mass % determined by EchoMRI at 28 weeks. (C) Average dry matter intake in grams. Data are averages of intake recorded for the diet group 3 times per week over the study. (D) Average energy intake in kilocalories (kcal). (E) Glucose measurements before the commencement of the diet. (F) Oral glucose tolerance test (OGTT) measured 28 weeks of diet commencement. (G) Quantification of glucose excursion after OGTT using area under the curve (AUC) measurements. (H) Livers were collected during sacrifice and weighed in milligrams (mg). (I) Insulin resistance measured during OGTT was determined by the homeostatic model of assessment of insulin resistance (HOMA-IR). Data are represented as mean ± SEM. Key: HF: high-fat. HS: high-sucrose. HFxHS: high-fat, high-sucrose interaction. HSD: high sucrose diet. HFD: high-fat diet. HFSD: high-fat, high-sucrose diet. F = f-statistic. p = *p*-value. HOMA-IR: homeostatic model assessment of insulin resistance. Log = logarithm to the natural base.

The anthropometric and physiological outcomes of the animals on the four diets after 30 weeks are shown in Figure 1. All data were analyzed using a two-way ANOVA type analysis, which tested for main effects of high sugar (HS) and high fat (HF) content, as well as interactions between the sugar and fat content (HFxHS). In the two obesity-related metrics of body weight and fat mass, there was no significant difference between the AIN93G diet (henceforth control) and high sucrose diet (HSD), but both high fat diet (HFD) and high-fat, high-sucrose diet (HFSD) showed significant increase and were not significantly different from each other (Figures 1A and 1B). These differences are only partially attributable to dietary intake. Animals maintained on HFD had small, but significantly greater average daily intake of dry matter with respect to the other three diet groups. Taking food intake (Figure 1C) and energy intake (Figure 1D) into account, there was a main effect of fat explaining the differences. In other metrics of metabolic health, we found basal glucose was significantly elevated at 30 weeks in HFD (Figure 1E), and glucose excursion after oral glucose load showed a trend to be higher in HFD and HFSD, albeit not significant (*p* = 0.06) (Figures 1F and 1G). Liver mass was significantly increased in both of the high-fat containing diets (Figure 1H), as was insulin resistance (Figure 1I) determined using the log homeostatic model of assessment for insulin resistance (logHOMA-IR).

### Cardiac structure and function showed diet-associated changes

**Figure 2:**
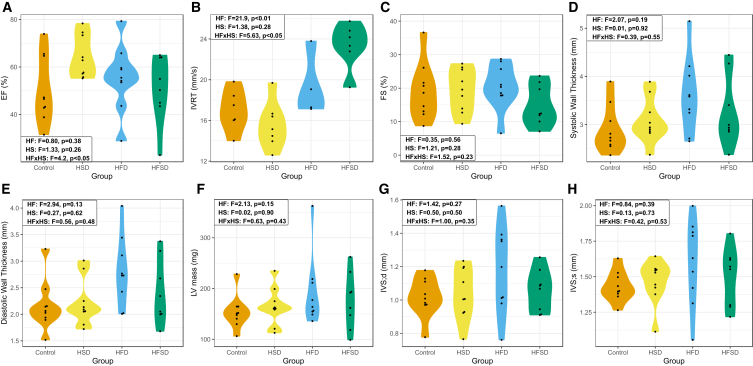
Diet-associated effects on cardiac structure and function (A) Left ventricular ejection fraction (EF) expressed as the percentage (%) of blood ejected from the left ventricle during each heartbeat was measured to assess cardiac systolic function. (B) Isovolumetric relaxation time (IVRT), an indicator of diastolic impairment was measured. (C) There was no change in fractional shortening (FS) between dietary groups. (D) Systolic and E. Diastolic wall thickness measurements were calculated from the measurements of LVAW and LVPW. (F) There was no significant difference in LV mass between dietary groups. (G) There was no significant difference in interventricular wall thickness in diastole (IVS,d); nor was there a significant difference in (H) inter-ventricular wall thickness in systole (IVS,s). Data are represented as mean ± SEM. Key: HF: high-fat. HS: high-sucrose. HFxHS: high-fat, high-sucrose interaction. HSD: high sucrose diet. HFD: high-fat diet. HFSD: high-fat, high-sucrose diet. F = f-statistic. p = *p*-value. EF = left ventricular ejection fraction. IVRT = isovolumetric relaxation time. FS = fractional shortening. LV = left ventricle. IVS, d = interventricular wall thickness in diastole. IVS, s = interventricular wall thickness in systole.

We next examined the cardiac structural and functional parameters in each dietary group (Figure 2). The effects of HSD were distinct from those of HFD and HFSD. This was seen for systolic function, as determined by left ventricular ejection fraction (EF), where lower EF indicates poorer systolic function; and diastolic function, as determined by isovolumic relaxation time (IVRT), where higher IVRT indicates diastolic impairment (Figures 2A and 2B). IVRT has been shown to be one of the most reliable murine indicators of diastolic impairment^20^ and we note there was also evidence of an interaction between fat and sucrose in the diet, with the HFSD leading to further augmented diastolic impairment over HFD, which was also detrimental in its own right. To understand the changes in systolic and diastolic function, we also examined whether there were structural changes, which often provide insight into the mechanism of functional change. However, despite the evidence of diastolic impairment, we did not see any corresponding thickening, or hypertrophy, of the left ventricle as indicated by increased thickness of the left ventricular walls in systole (Figure 2E), and diastole (Figure 2F), despite a trend in the HFD group. In a similar vein, there was no significant change in LV mass (Figure 2G), interventricular wall thickness in systole (Figure 2H), or in diastole (Figure 2I), despite a trend in the HFD group.

### Select metabolites had consistent changes across organ pools, except for cecum

**Figure 3:**
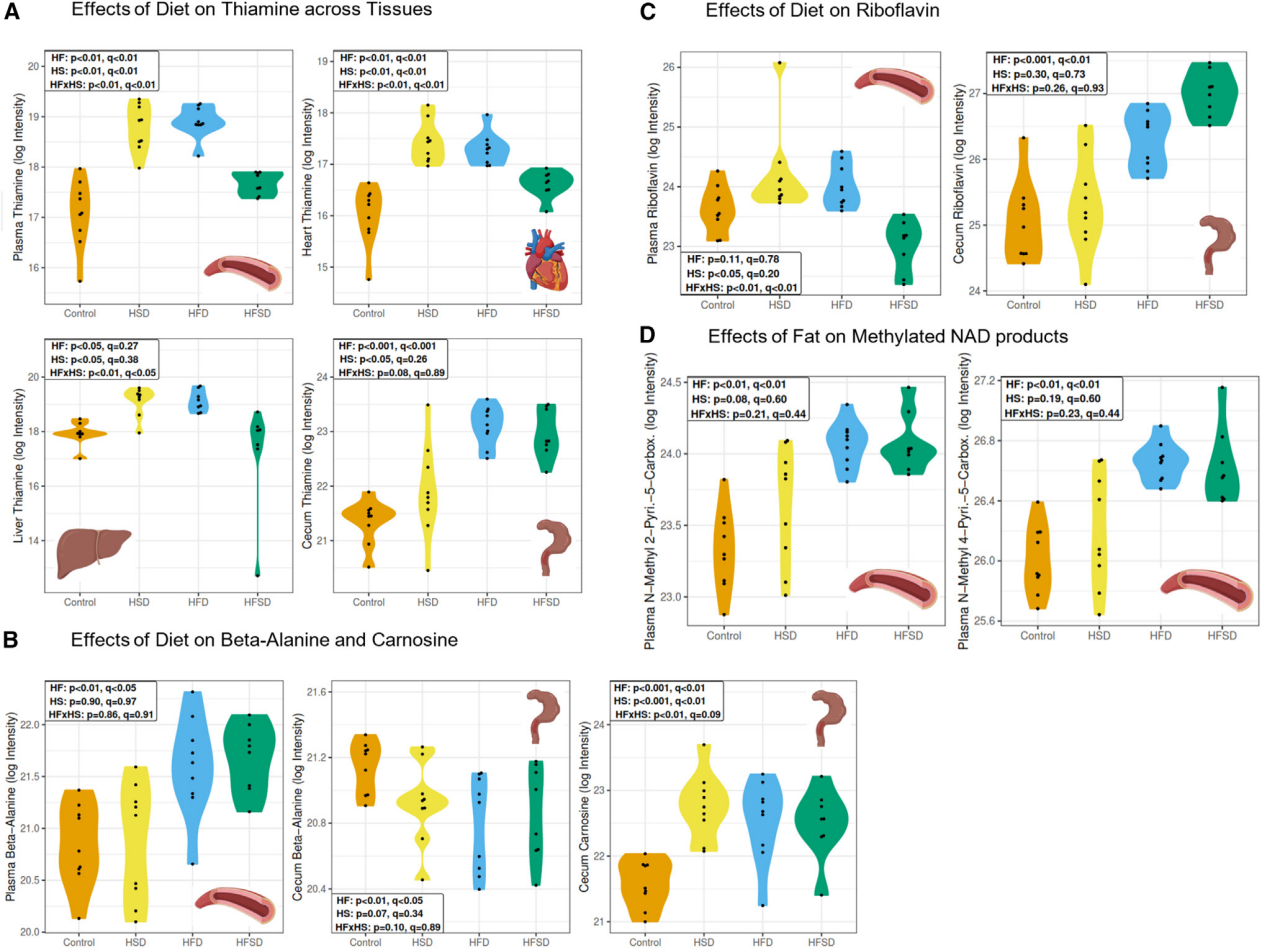
Changes in thiamine, beta-Alanine, carnosine, riboflavin, and methylated NAD products were evident across multiple organs (A) Thiamine was significantly elevated in the plasma, heart, and liver of both HSD and HFD groups, and also in the cecum of the HFD group. (B) Beta-alanine was significantly elevated in the plasma and significantly lower in the cecum of those on high-fat diets. Carnosine, a metabolite of beta-alanine, was significantly elevated in the cecum of HSD and HFD. (C) Plasma levels of riboflavin demonstrated a fat-sucrose interaction effect, whereas, in the cecum, riboflavin was elevated by high-fat diets. (D) Two-methylated NAD products, N-methyl-2-pyridone-5-carboxamide (2PY) and N-methyl-4-pyridone-5-carboxamide (4PY), were significantly elevated in the plasma of high-fat diet groups. Key: HF: high-fat. HS: high-sucrose. HFxHS: high-fat, high-sucrose interaction. HSD: high sucrose diet. HFD: high-fat diet. HFSD: high-fat, high-sucrose diet. q = q-statistic (i.e., adjusted *p*-value). p = *p*-value. NAD = nicotinamide adenine dinucleotide. Log intensity = log transformation of the area under the curve of intensity reported on the LC-MS/MS.

**Figure 4:**
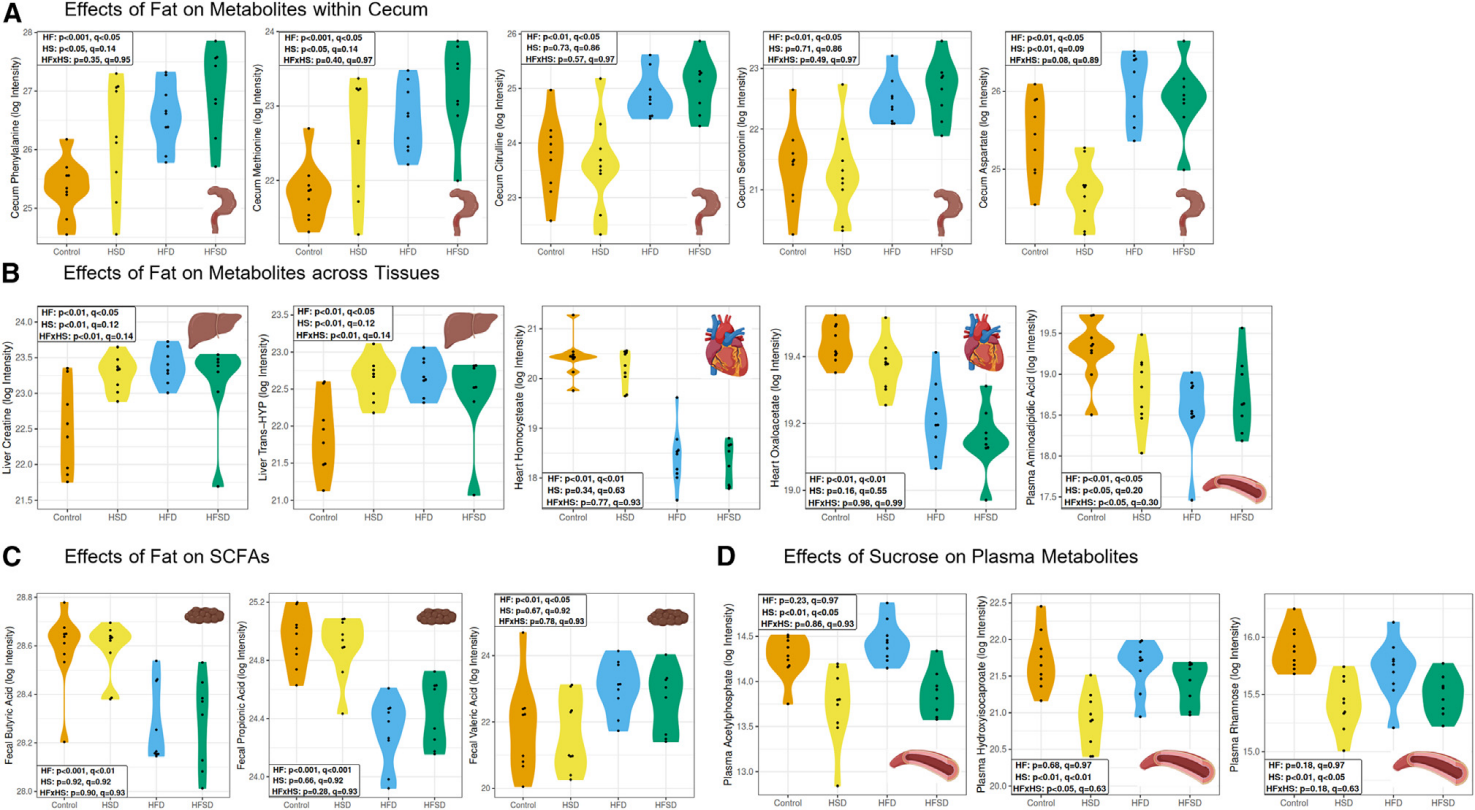
High-dietary fat had wide-ranging effects on metabolites in the cecum, liver, heart, plasma, and feces; Sucrose affected a smaller range of plasma metabolites (A) High-fat diets significantly elevated cecum phenylalanine, methionine, citrulline, serotonin, and aspartate. (B) High-fat diets significantly elevated liver creatine and *trans*-hydroxy-*l*-proline (trans-Hyp), decreased heart homocysteate and oxaloacetate, and decreased plasma aminoadipic acid. (C) High-fat diets decreased fecal levels of short-chain fatty acid butyric acid, propionic acid, and elevated levels of short-chain fatty acid valeric acid. (D) High-sucrose diets significantly decreased plasma levels of acetylphosphate, hydroxyisocaproate, and rhamnose. Key: HF: high-fat. HS: high-sucrose. HFxHS: high-fat, high-sucrose interaction. HSD: high sucrose diet. HFD: high-fat diet. HFSD: high-fat, high-sucrose diet. q = q-statistic (i.e., adjusted *p*-value). p = *p*-value. *Trans*-HYP = *trans*-hydroxy-*l*-proline. Log intensity = log transformation of the area under the curve of intensity reported on the LC-MS/MS.

Metabolism is tightly regulated to adaptively respond to changes in nutrient supply over time and preserve homeostasis. In any one compartment of the body, the observed metabolite pool will reflect cumulative effects of, *inter alia*, food intake and digestion, translocation of molecules from the gut to systemic tissues, and cell/tissue specific metabolic activity. In characterizing the diet-induced responses we assessed metabolite pools in multiple body compartments (Figures 3 and 4).

The experimental diets (AIN93G chow, HSD, HFD, and HFSD) were designed to have matching amounts of vitamins and minerals (Table S1). It is therefore noteworthy that several vitamins showed significant differences. Thiamine was significantly elevated in the plasma, heart, and liver of both HSD and HFD groups, and also in the cecum of the HFD group (Figure 3A). There was also evidence of an interaction between sucrose and fat on levels of thiamine in the plasma, heart, and liver, but not in the cecum (Figure 3A). Plasma levels of riboflavin demonstrated a fat-sucrose interaction effect, whereas in the cecum, riboflavin was elevated by high-fat diets (Figure 3C). Two-methylated nicotinamide adenine dinucleotide (NAD) products, N-methyl-2-pyridone-5-carboxamide (2PY) and N-methyl-4-pyridone-5-carboxamide (4PY), were significantly elevated in the plasma of high-fat diet groups (Figure 3D). These methylated NAD products were recently reported to result from spillover of the NAD pool, and linked to increased risk for heart attack, stroke, and major adverse cardiovascular outcomes by promoting vascular inflammation.^21^

Beta-alanine was significantly elevated in the plasma in those on high-fat diets, and significantly lower in the cecum in high-fat diet groups (Figure 3B). Carnosine, a metabolite of beta-alanine, was significantly elevated in the cecum of HSD and HFD (Figure 3B).

The two high-fat diet groups also had significant elevations in liver creatine and liver *trans*-hydroxyl-*l*-proline; they had decreased levels of heart homocysteate, and heart TCA intermediate oxaloacetate (Figure 4B). High-fat diet groups had significantly decreased levels of plasma aminoadipic acid, and evidence of a fat-sucrose interaction on plasma aminoadipic acid levels (Figure 4B). The HSD group had significantly lower levels of plasma acetylphosphate, hydroxyisocaproate, and rhamnose (Figure 4D).

### Consistent fat and sucrose effects across metabolite pools

Microbial metabolites have been implicated in both protective and harmful metabolic outcomes. Diets high in saturated fat have previously been reported to have strong effects on microbial activity via changes in bile profile.^5^ In our study, significant cecal metabolite pool changes related to amino acid metabolism were seen for both HFD and HFSD diets. These included elevated levels of phenylalanine, methionine, citrulline, serotonin, and aspartate with respect to control and HSD groups (Figure 4A). This is consistent with the change in microbial metabolite production, although to our knowledge none of these have been specifically linked with outcomes. The most widely identified microbial metabolites with health consequences are short chain fatty acids (SCFAs). Indeed, SCFAs supplied in drinking water or via diet supplements have been implicated in protection against hypertension and a recent clinical trial assessing the potential for food supplements increasing acetate and butyrate supply to protect against hypertension found evidence for effectiveness.^22^ The HFD and HFSD diets did show a noteworthy impact on butyrate, propionate and valerate, whereby butyrate and propionate were both decreased on these diets, and valerate increased (Figure 4C).

### Diet-associated changes in the gut microbiome

**Figure 5:**
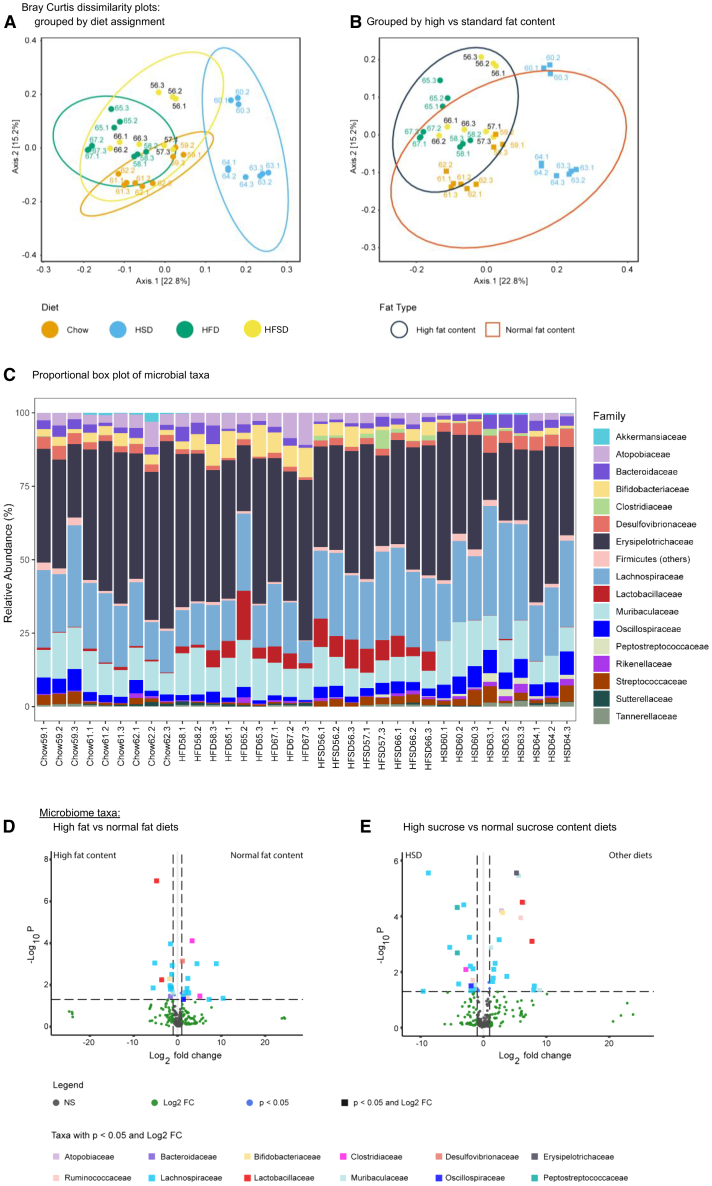
Differences in microbial community states may reflect selection mechanisms related to inclusion of lard and displacement of starch in diets (A) Bray–Curtis dissimilarity plots illustrating the distinct separation of HFD, with intra-group clustering according to cage number. (B) Bray–Curtis dissimilarity plot by high vs. standard fat content, showing overlap and distinct clustering according to cage number. (C) Proportional boxplot of microbial taxa showing enrichment of lactobacillaceae (red) and bifidobacteriaceae (yellow) in HFD; enrichment of desulfovibrionaceae (orange), bacteroidaceae (purple) in HSD, with a decrease in atopobiaceae (lavender). (D) Volcano plot illustrating significant taxa changes in high-fat vs. normal fat diets. (E) Volcano plot illustrating significant taxa changes in high-sucrose vs. normal sucrose diets. Key: HF: high-fat. HS: high-sucrose. HSD: high sucrose diet. HFD: high-fat diet. HFSD: high-fat, high-sucrose diet. P = *p*-value. Log_2_ = logarithm to the base 2. Log_10_ = logarithm to the base 10. FC = fold-change.

We next examined the effects of diet manipulations on the gut microbiome. Analysis of beta diversity patterns revealed significant effects of both diet treatment and caging on community structure (Figures 5A and 5B). Each diet group had eight to nine animals in three separate cages and, within the diet group, microbial communities of animals in the same cage were significantly more similar. This effect was strongest for the HSD group. Overall, diet effects were sufficiently selective that independently caged mice on the same diet had more similar microbiomes than those on distinct diets (Figures 5A and 5C).

From the microbial ecology perspective, our diet manipulations have two broad postulated axes of impact: (1) Displacement of starch as the primary source of microbially accessible carbohydrate (MAC) by more host accessible nutrients that are efficiently absorbed in the small intestine (sucrose, fat or protein). This axis was strongest in the HSD diet where starch was reduced from 40% to 9.69% through replacement with sucrose, and; (2) Environmental change due to the introduction of high levels of saturated fat (15% lard) and increased proportion of protein (increased from 20 to 25%). This axis was common between HFD and HFSD, with these two diets also differing in starch displacement, which was greatest in HFSD. There is evidence that these distinct impact axes are reflected in the community structure with microbiomes on the two diets containing lard and increased casein (HFD and HFSD) clustering together and the extremely low MAC diet (HSD) being the outlier (Figure 5B).

The distribution of major taxa at the family level across the four diet groups is shown in Figure 5C. Enrichment of *Lactobacillaceae* (red) and *Bifidobacteriaceae* (yellow) on the two lard+casein diets (HFD and HFSD) relative to HSD is obvious. The *Lactobacillaceae* enrichment on these diets is consistent with the selection for casein utilization. Relative to the lard-containing diet groups, the extremely low MAC diet (HSD) is characterized by relative enrichment of *Desulfovibrionaceae* (orange), and *Bacteroidaceae* (purple), and a decrease in *Atopobiaceae* (lavender) (Figure 5C). Volcano plots illustrate significant changes of taxa in high-fat vs. normal fat diets (Figure 5D) and taxa in high-sucrose vs. normal sucrose diets (Figure 5E).

### Multi-omic factor analysis (MOFA) of microbiota with metabolites from cecum and plasma reveals key positive and negative correlations across molecular layers

**Figure 6:**
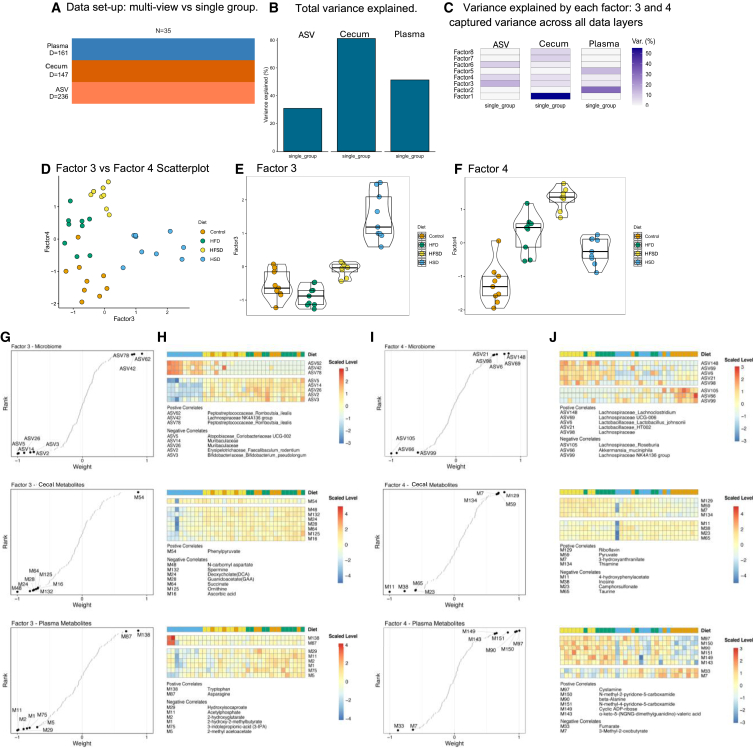
MOFA multi-omic analysis of microbiota with metabolites from cecum and plasma (A) The design for implementation of the MOFA method. (B) Total variance explained by microbiome ASV, cecal metabolite, and plasma metabolites. (C) Variance in each data layer was explained by 8 factors; factors 3 and 4 explained most variance across all three. (D) Factor 3 vs. Factor 4 scatterplot demonstrates distinct separation of the 4 dietary groups. (E) Factor 3 was most different in HSD. (F) Factor 4 was different in all three experimental groups, but most different in HFSD. (G) For Factor 3, weight and rank of microbiome ASVs (Top), cecal metabolites (Middle), and plasma metabolites (Bottom). (H) Heatmap of Factor 3 scaled level per diet of ASV (Top), cecal metabolites (Middle), and plasma metabolites (Bottom) with top candidate correlations listed below in each case. (I) For Factor 4, weight and rank of microbiome ASVs (Top), cecal metabolites (Middle), and plasma metabolites (Bottom). (J) Heatmap of Factor 4 scaled level per diet of ASV (Top), cecal metabolites (Middle), and plasma metabolites (Bottom) with top candidate correlations listed below in each case. Key: MOFA: multi-omic factor analysis. ASV: amplicon sequence variant. HSD: High-sucrose diet. HFD: high-fat diet. HFSD: high-fat, high-sucrose diet.

We next used the MOFA method^23^ to perform a multi-omic analysis of microbiota data along with metabolites from cecum and plasma (Figure 6). To do this analysis, we compared multi-view vs. single-group analysis (Figure 6A), as described.^23^ The total variance explained was greatest for cecal metabolites, followed by plasma metabolites, and then microbiota amplicon sequencing variants (ASVs) (Figure 6B). There were relevant factors explaining variance in the three data layers, with factors 3 and 4 capturing variance across the three sets (Figure 6C). Plotting Factor 3 vs. Factor 4, excellent separation of the 4 dietary groups was seen (Figure 6D). The similarity of the variance explained by Factor 3 in each dietary group is plotted in Figure 6E, and HSD displayed the greatest divergence in Factor 3. For Factor 4 (Figure 6F), all experimental diets are separate from the control diet, with the HFSD diet being the most different. Next, we outline the correlation of factors 3 and 4 at the microbiome, cecal metabolite, and plasma metabolite level (Figures 6G–6J). The microbiota (top panels), cecal metabolites (middle panels), and plasma metabolites (bottom panels) are shown in each case.

ASV62, 42, and 78 (*Peptostreptococcaceae* and *Lachnospiraceae*) were most positively correlated with factor 3, the principal component most divergent in the high sucrose diet (upper panels, Figures 6G and 6H). ASVs comprising *Atopobiaceae, Muribaculaceae, Erysipelotrichaceae,* and *Bifidobacteriaceae* were most negatively correlated with factor 3. ASVs comprising *Lachnospiraceae* species were the most positively correlated with factor 4, the principal component most divergent in the dual HFSD (lower panels, Figures 6I and 6J). ASVs comprising certain *Lachnospiraceae* and *Akkermansia* species were most negatively correlated with factor 4.

Phenylpyruvate was the most strongly associated cecal metabolite with factor 3, and N-carbamoyl aspartate was the most negatively correlated with factor 3 (middle panels, Figures 6G and 6H). Riboflavin was the cecal metabolite with the most significant positive correlation with factor 4, whereas 4-hydroxyphenylacetate was the most negatively associated cecal metabolite with factor 4 (middle panels, Figures 6I and 6J).

With regards to plasma metabolites, tryptophan and asparagine were most significantly positively correlated with factor 3, whereas hydroxyisocaproate and acetylphosphate were the most negatively correlated plasma metabolites with factor 3 (bottom panels, Figures 6G and 6H). Plasma cystamine was the most positively correlated metabolite with factor 4, whereas fumarate and 3-methyl-2-oxobutyrate were the most negatively correlated metabolites with factor 4 (bottom panels, Figures 6I and 6J).

### Correlation across all data layers reveals 4 distinct pheno-signatures: one comprising variables inversely associated with cardiometabolic disease, two associated with detrimental metabolic effects, and another inversely associated with cardiac contractile function

**Figure 7:**
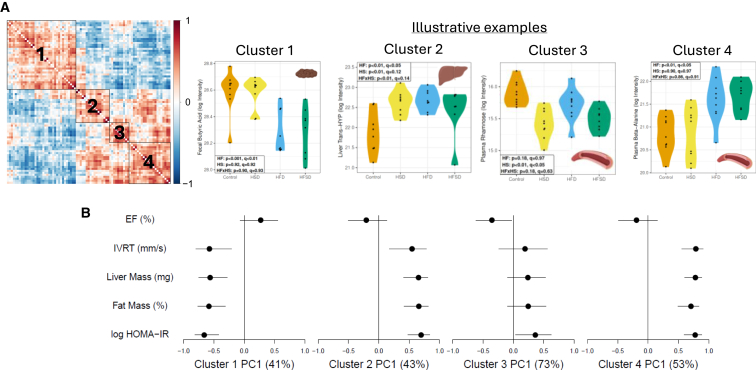
Correlation across all data layers reveals 4 distinct pheno-signatures: one comprising variables positively correlated with cardiometabolic phenotypes, two negatively correlated with cardiometabolic phenotypes, and one negatively correlated with cardiac contractile function (A) Correlation matrix identifies 4 distinct clusters. Illustrative examples for each cluster are given. Cluster 1: fecal butyric acid. Cluster 2: Liver *trans*-hydroxyl-proline. Cluster 3: Plasma rhamnose. Cluster 4: Plasma beta-alanine. (B) Association of each data cluster with cardiac and metabolic phenotypes. A complete list of the components of each cluster is provided in Table S2. Key: EF = ejection fraction. IVRT = isovolumetric relaxation time. Mm/s = millimeter/s. Mg = milligram. % = percentage. p = *p*-value. q = q-statistic (i.e., adjusted *p*-value). Log HOMA-IR = natural logarithm of the homeostatic model assessment of insulin resistance. PC = principal component. Log intensity = log transformation of the area under the curve of intensity reported on the LC-MS/MS.

For those metabolites and microbes with a statistically significant effect of diet, we next correlated metabolomic data (across plasma, gut contents, and all organs) and gut microbiota at the level of individual animals. The resulting correlation matrix identifies 4 distinct clusters (Figure 7A). We then performed correlation analysis of the first principal component of the 4 clusters with cardiac, physiological, and metabolic phenotypes (Figure 7B). The principal components explained between 41 and 73% of the variance in each cluster. This analysis identified that variables in Cluster 1 are negatively correlated with metabolic disease; Clusters 2 and 4 positively with metabolic disease; and Cluster 3 negatively with cardiac contractile function. The components of each cluster are listed in Table S2. Summarily, cluster 1 contains a depletion of putatively protective factors that are associated with 26 bacterial ASVs including most of those classified to genera *Roseburia*, *Lachnoclostridium* (both in *Lachnospiraceae*) and *Romboutsia* (*Peptostreptococcaceae*). We postulate activity (or abundance) of these microbe groups may potentiate these factors. Cluster 2 is associated with increased metabolic risk and comprised a highly correlated group of metabolites including branched-chain amino acids, methionine, and thiamine, along with phylogenetically distinct microbe ASVs assigned to unclassified *Lachnospiraceae, Muribaculaceae*, and *Bacteroides*. Cluster 4 is also associated with detrimental metabolic effects, containing plasma beta-alanine and cecal riboflavin, citrulline, serotonin, and aspartate, along with ASVs assigned to *Lactobacillus* and *Bifidobacterium*. Cluster 3, associated with impaired cardiac contractile function, is composed of a smaller group of plasma metabolites such as acetylphosphate, hydroxyisocaproate, and rhamnose, with *Faecalibaculum* and multiple ASVs assigned to *Muribaculaceae*. Interestingly, the metabolites in this group are decreased in diets with elevated sugar content.

## Discussion

As expected, high-fat diets led to an accumulation of fat mass, hepatomegaly, and corresponding elevations in fasting glucose and HOMA-IR. Consistent with these changes, high-fat diet groups (HFD and HFSD) displayed cardiac diastolic dysfunction without a significant change in cardiac structure. These cardiac findings are typical of left ventricular mass-independent diastolic impairment seen clinically in obesity.^24^ Notably, both the HFD and HFSD groups displayed marginally elevated dry-weight food intake, and thanks to the high energy content of these diets, substantially elevated energy intake. Hence, what we refer to as high-fat effects may well be attributed to energy imbalance rather than a toxic metabolic effect of fat *per se*.

We observed changes in metabolites that were consistent across organs. For example, thiamine was elevated in both HSD and HFD in the plasma, heart, and liver, and demonstrated a fat-sucrose interaction in all three sites. However, in the cecum, thiamine was elevated in high-fat diets and not HSD. However, there was a wide range of changes in thiamine levels in the cecum within the HSD group, with some displaying increases and some not. This suggests that other factors were interacting with thiamine levels in the cecum of HSD mice. Thiamine is an important coenzyme in glucose metabolism.^25^ It is deficient in the obese, and purported to contribute to inefficient glucose metabolism and buildup of anerobic intermediates such as lactate.^26^ However, our data suggests an obesity-independent effect, with equal enrichment in the metabolite pools of HSD and HFD, but the underlying reason remains unclear. In our data, cecal elevation of thiamine, suggesting increased excretion via the gut, on high-fat diets is consistent with the reported deficiency of circulating thiamine in obesity, as both high-fat diets led to obesity. However, our data suggests that there are other macronutrient dependent factors at play, which warrant further investigation.

Plasma and cecal beta-alanine levels displayed an inverse pattern, whereby HFD and HFSD groups had elevated plasma beta-alanine levels, but decreased cecal levels, potentially suggesting increased cecal permeability for beta-alanine in mice on high-fat diets. Carnosine, a dipeptide of beta-alanine and histamine,^27^ had cecal levels that were inverse to those of cecal beta-alanine, which offers a countervailing hypothesis— that the reason for lower cecal beta-alanine is due to local conversion to carnosine in the gut. Carnosine, unlike beta-alanine, was also affected by HSD. Carnosine is formed from beta-alanine and L-histidine in the liver,^28^ and both beta-alanine and carnosine are readily absorbed via the human small intestine by specific transporters,^28^ but little is known about the conditions that govern breakdown of carnosine to beta-alanine in the gut. Carnosine is purported to be a compensatory protective factor in metabolic syndrome, with some evidence suggesting carnosine increases insulin secretion, pancreatic beta cell mass, antioxidant effects, scavenging of reactive carbonyl species, and leads to reduced formation of advanced lipoxidation and glycation end products.^29^^,^^30^ Together, it is highly plausible that high-fat diets lead to greater absorption of beta-alanine from the gut. The sucrose and fat effects on carnosine are more difficult to interpret with the available data, as there are likely both hepatic and extra-hepatic enzymatic effects on carnosine levels via carnosinases and carnosine synthetase.^28^ Despite the significant increase in carnosine levels in the gut on HSD and high-fat diets, we did not see a significant change in the plasma.

Vitamin B2, or riboflavin, was significantly elevated in the plasma of HSD and HFD groups, with a clear negative interaction displayed in dual HFSD, which had lower plasma levels. Cecal riboflavin, on the other hand, displayed a positive interaction, with the highest levels in the dual HFSD. Together, the plasma-cecal riboflavin levels again displayed inverse patterns, suggesting potential transfer across the gut wall, with evidence of fat-sucrose interaction. Riboflavin, a water-soluble vitamin, is mainly involved in energy metabolism in the forms of flavin mononucleotide (FMN) and flavin adenine dinucleotide (FAD).^31^ Most relevant to our study parameters is the reported synergistic role of riboflavin, when deficient, with high-fat diets in promoting metabolic diseases such as non-alcoholic fatty liver disease (NAFLD).^32^ However, our data encourages consideration of a non-obesogenic, high-sucrose effect on riboflavin, as well as strong sucrose-fat interactions evident in the circulation and gut.

Recent work has demonstrated residual cardiac risk (and vascular inflammation) at the population level stemming from dietary niacin supplementation leading to NAD pool spillover to two methylated waste products: N1-methyl-2-pyridone-5-carboxamide (2PY) and N1-methyl-4-pyridone-3-carboxamide (4PY).^21^ Intriguingly, in our study where there was no niacin supplementation, high-fat containing diets displayed significantly elevated plasma levels of both methylated products, 2PY and 4PY. This may be another mechanism by which high-fat diets cause vascular injury.

High-fat diets increased cecal levels of several metabolites in a consistent fashion, including phenylalanine, methionine, citrulline, serotonin, and aspartate. However, these changes were not propagated outside the gut, and it is unclear if these gut changes have any systemic effects. HFD mice had significant elevations in liver creatine and liver *trans*-hydroxyl-*l*-proline. Creatine is a non-essential amino acid.^33^ The first step of synthesis occurs in the kidney, whereby glycine and arginine are metabolized by glycine amidinotransferase (AGAT) to form ornithine and guanidinoacetate.^34^ The second step in the liver is catalyzed by N-guanidinoacetate methyltransferase (GAMT), whereby a methyl group from S-adenosyl methionine, a methyl donor, is transferred to guanidinoacetate.^34^ This step produces creatine and S-adenosyl homocysteine. Synthesis of creatine is regulated by several factors, including hormonal (testosterone, estrogen, growth hormone, and thyroxine regulation of AGAT), levels of intermediate ornithine, product creatine, and activity of AGAT. However, likely the most relevant for our study is the regulation of creatine transporter SLC6A8 by the AMP-activated protein kinase.^35^ This protein kinase is a sensor of cellular energetic status and is known to couple substrate transport to capacity of cells to yield energy. The AMP-activated protein kinase is activated in conditions of energy depletion. It was shown by Li et al.^35^ to inhibit SLC6A8 via the mammalian target of rapamycin (mTOR) pathway. Therefore, when there is energy depletion, there is less ATP to require buffering by creatine, and therefore uptake of creatine by SLC6A8 is reduced. However, in high-energy states consequent upon HFD, there is no limitation on creatine generation, as seen in the liver in our study. Elevation of liver *trans*-hydroxyl proline on HFD may represent increased proline hydroxylation seen in hepatic insulin resistance.^36^

Intriguingly, there was a dramatic decrease in cardiac levels of homocysteate, which is a sulfur-containing glutamic acid analog and reported as a potent NMDA receptor agonist. It is also related to homocysteine, reported to be a promoter of free-radical oxidative damage and a vascular risk factor.^37^ The TCA intermediate, oxaloacetate, was also significantly depleted in the heart of HFD and HSD groups; there was a greater effect in HFD than HSD, with an interaction in the dual diet leading to greater suppression of cardiac oxaloacetate. Reduced oxaloacetate may suggest less efficient energy metabolism and flux through the TCA cycle. Aminoadipic acid, a lysine product associated with diabetes and known to be an insulin secretagogue,^38^ was lower in the heart of the experimental diets, especially the HFD group. We previously demonstrated that elevated aminoadipic acid was associated with incident diabetes mellitus, and in the prodromal phase may be a compensatory mechanism to upregulate insulin secretion.^38^ However, this study represents a far later stage of dysglycemia, after 30 weeks of experimental dietary overnutrition, and as such, one would not expect to see the same early counter-regulatory response in aminoadipic acid.

Fecal levels of SCFAs changed most on high-fat containing diets, with a significant decrease in butyrate and propionate. Since these diets had reduced starch levels, this is broadly consistent with previous reports of diets with low MAC content (dietary fiber, resistant starch) being less able to support bacteria fermentation activity leading to SCFAs.^39^ We also note that these diets had increased dietary casein and high levels of saturated fat. Saturated fat has been found to induce bile profiles that select against butyrogenic bacteria and the ratio of protein to carbohydrate can also drive distinct microbial community outcomes. Another SCFA, valeric acid, was significantly increased in these diets. Since such branched-chain fatty acids are typically the products of protein fermentation, this is consistent with the increased protein in these diets having also influenced microbiome outcomes.

High sucrose diets were also associated with several metabolite changes. For example, HSD decreased plasma concentrations of acetylphosphate, hydroxyisocaproate, and rhamnose. Acetyl phosphate is a high-energy intermediate of the phosphotransacetylase-acetate kinase pathway, playing a role in post-translation protein acetylation^40^; its relevance in this context is not immediately clear. Hydroxyisocaproate is an end product of leucine metabolism in human tissues, known to accelerate lipid peroxidation and indicative of oxidative stress,^41^ and at least in this respect, high sucrose diet promotes lower levels of this deleterious molecule. Rhamnose is a naturally occurring deoxy sugar, a part of plant cell wall polysaccharides, and present in bacteria but not mammals. As a non-absorbable monosaccharide, it has previously been reported to promote the generation of the SCFA propionate.^42^^,^^43^ It is not actively absorbed in the small intestine and reaches the colon intact where it is fermented by a limited microbial community.^44^ However, our data show that it is high-dietary fat that leads to least fermentation of fecal propionate, without an evidentiary effect on rhamnose. As fecal propionate was unaffected by HSD, it may rather suggest that HSD prevents the gut transit and/or absorption of rhamnose into the circulation, although further study is required to elicit the exact mechanisms.

Our cluster analysis revealed 4 groups of co-regulated metabolites and microbiota across all locations. Cluster 1 represents metabolites and microbiota that were inversely correlated with the fat-dependent phenotypes of cardiac diastolic impairment, hepatomegaly, fat mass, and insulin resistance (HOMA-IR). Examples of components of this cluster include plasma aminoadipic acid, fecal SCFAs butyric acid and propionic acid, cardiac homocysteate, and microbiota *Lachnospiraceae Roseburia*. However, the interaction of these specific metabolites and gut microbiota in this context indicates a potential fat-microbe feedback counter-regulation of fat-dependent phenotypes.

Cluster 2 was positively correlated with these fat-dependent phenotypes. Components of cluster 2 included liver creatine, liver trans hydroxyproline, cecal phenylalanine, cecal thiamine, and *Lachnospiraceae* and *Lachnoclostridium* microbiota. These likely represent collective potentiators of fat-dependent cardiometabolic perturbation.

Cluster 3 comprised components driven by HSD, which actually led to a non-significant increase in systolic (contractile function) of the heart. The components of this cluster were inversely correlated with cardiac contractile function, and the first principal component of this cluster remarkably explained 73% of the variance in this phenotype. Components of cluster 3 included plasma rhamnose, acetylphosphate, hydroxyisocaproate, and microbiota *Muribaculaceae*. The strong coregulation of this small group presents an opportunity to probe counter-regulatory responses to sucrose over-supply using these select intermediates.

Cluster 4 comprised components that were upregulated by HFD, much like cluster 2. Cluster 4 was more strongly associated with these phenotypes than cluster 2, especially cardiac diastolic impairment. Cluster 4 composed cecal metabolites such as riboflavin, citrulline, serotonin, and aspartate. Plasma beta-alanine, 2PY, and 4PY were also in cluster 4, as was microbiota *Lachnospiraceae Blautia coccoides*. 2PY and 4PY are highly topical spillover metabolites from the NAD pool, recently reported to represent a considerable proportion of residual, unexplained cardiovascular risk and markers of incident myocardial infarction.^21^ To our knowledge, we are the first to report an association of HFD with these increased terminal products of niacin and mediators of vascular inflammation. The potential role of the above cecal metabolites in these cardiometabolic phenotypes also warrants further study.

### Limitations of the study

This is an experimental study using defined diets and an inbred mouse strain. As such, the data reveal associations in a specific physiological context and the extent to which interpretations are generalizable is limited. The microbiome data are based on amplicon sequencing data that provide high taxonomic resolution in the inbred mouse context. Microbial physiological traits are inferences based on attribution of rDNA sequences to taxonomic database. Associations reported here and the postulated mechanisms should be viewed as plausible explanations.

As an experimental study, our results have a high degree of internal validity, though this degree of experimental control may come at the cost of generalizability. First, our experiment was undertaken in male C57BL6/J mice, which is the most common pre-clinical model of metabolic disease. However, in both pre-clinical models and in human diet can have sex- and genotype-specific effects on metabolic health and disease.^45^^,^^46^ It remains to be seen if our results translate across these contexts. Although our metabolomics platform contains sentinels across most metabolic pathways, more untargeted approaches might review further insights.

### Conclusion

We herein outline the factors, collinear and divergent, that are modulated across different organ pools, and how they relate to cardiometabolic phenotypes, in response to fat and sucrose dietary over-supply. We identify several unique molecular changes, such as spillover of methylated products from the NAD pool —known to cause vascular inflammation—with dietary fat oversupply.

Furthermore, we reveal distinct groups of co-regulated metabolites, SCFAs, and microbiota that advance insight into development of dietary-induced cardiometabolic phenotypes. For example, the observed alterations in thiamine and riboflavin levels across the different tissues suggest significant interactions between macronutrients and metabolic pathways, highlighting potential obesity-independent effects.

We also highlight several different metabolite and microbiome candidates that warrant further study as mediators of Western dietary effects in cardiometabolic disease. The inverse correlations observed among certain metabolites such as beta-alanine, carnosine, and fat-dependent phenotypes suggest potential counter-regulatory mechanisms that could inform future therapeutic strategies.

## Resource availability

### Lead contact

Further information and requests for resources and reagents should be directed to and will be fulfilled by the lead contact, Prof John O’Sullivan (john.osullivan@sydney.edu.au).

### Materials availability

This study did not generate new unique reagents.

### Data and code availability

• Raw data from Figures 1, 2, 3, 4, 5, 6, and 7 were deposited on Mendeley at https://doi.org/10.17632/6z3z7p7y8r.1.
• All original code has been deposited at Zenodo at https://doi.org/10.5281/zenodo.14606894 and is publicly available as of the date of publication.
• Any additional information required to reanalyze the data reported in this work paper is available from the lead contact upon request

## Acknowledgments

We thank SydneyMS for providing the instrumentation used in this study. We thank the Ramaciotti Center for Genomics at University of New South Wales for their help in analyzing the fecal samples for microbiome analysis. J.O.S. is supported by a National Heart Foundation Future Leader Fellowship (104853), MRFF (2024161), and NSW EMCR award. Y.C.K. is supported by an NSW Cardiovascular Collaborative Grant (OHMR23-251985) and a National Heart Foundation Future Leader Fellowship (107180).

## Author contributions

J.O.S. and Y.C.K. conceived the study. Y.C.K. and J.O.S. designed experiments. Y.C.K. and R.P.L. performed experiments. Y.C.K., R.P.L., A.S., Z.B., and A.H. were involved in data analysis. R.P.L., A.H., and J.O.S. wrote the manuscript, and the final version of the manuscript was reviewed and approved by all authors.

## Declaration of interests

The authors declare there are no conflicts of interest.

## STAR★Methods

### Key resources table

**Table:** 

REAGENT or RESOURCE	SOURCE	IDENTIFIER
**Chemicals, peptides, and recombinant proteins**		

EDC (1-ethyl-3-(3-dimethylaminopropyl)carbodiimide hydrochloride)	Thermo-fisher	Cat#22980
Girard’s reagent T (GT)	Sigma-Aldrich	Cat#G900
Sodium Acetate	Sigma-Aldrich	Cat#S2889
Sodium Propionate	Sigma-Aldrich	Cat#P1880
Sodium Butyrate	Sigma-Aldrich	Cat#B5887
Valeric Acid	Sigma-Aldrich	Cat#240370
Hexanoic/Caproic Acid	Sigma-Aldrich	Cat#21529
Acetonitrile	Thermo Fisher Scientific	Cat#325730025
Methanol	Thermo Fisher Scientific	Cat#325740025
Formic acid	Thermo Fisher Scientific	Cat# 28905
Valine-d8	Sigma-Aldrich	Cat# 486027
Phenylalanine	Sigma-Aldrich	Cat# DLM-372-PK
Thymine d4	Sigma-Aldrich	Cat# 487066

**Deposited data**		

Raw and analyzed data	This paper; Mendeley data	https://doi.org/10.17632/6z3z7p7y8r.1
Raw metabolomics data	This paper; NMDR	ST003253 ST003252

**Critical commercial assays**		

Ultra-Sensitive Mouse Insulin ELISA Kit	Crystal Chem Inc	Cat#90080
FastDNA™ SPIN Kit for Feces	MP Biomedicals Australiasia	Cat#6570200

**Experimental models: Organisms/strains**		

Mouse: C57BL6/J	Australian Resources Centre (ARC)	

**Software and algorithms**		

FUJIFILM VisualSonics	VisualSonics	VevoLAB
R	The R Foundation for Statistical Computing	V 4.3.1

### Experimental model and study participant details

#### Animals and husbandry

36 male C57BL6/J mice were obtained from the Australian Resource Centre (WA, Australia) at 6 weeks old and housed at the Charles Perkins Centre at the University of Sydney under a 12 hour light: dark cycle (protocol 2017/1294). Animals were housed three per cage in standard approved cages. Mice were allowed 2 weeks acclimatization before experimentation began. At 8 weeks of age, mice were allocated to four different diets consisting of a control diet (CHOW), a high-sucrose diet (HSD), a high-fat diet (HFD), and a high-fat high-sucrose (western) diet (HFSD). All experimental diets were custom designed and manufactured in dry, pelleted form by Specialty Feeds (WA, Australia). Both the control diet and high-sucrose diet were isocaloric (14.4 MJ/Kg) and matched in total calculated net metabolizable energy (NME) from protein (19%) carbohydrate (61%) and fat (20%), while the high-fat diet and high-fat high sucrose diet were isocaloric (18.1 MJ/Kg) and matched in the total calculated NME from protein (19%) carbohydrate (36%) and fat (45%). Detailed compositions of the diets are listed in Table S1. Mice had *ad libitum* access to their food and autoclaved water. Body weights were recorded weekly and food intake (consumption per cage) was measured three times a week throughout the study. Mice from each dietary group were euthanized using overdose intraperitoneal injections of pentobarbital (Nembutal®, Abbott Laboratories, North Chicago, IL, USA; 25 mg/mL, 75 mg/kg) as approved by the Institutional Animal Ethics Committee at the University of Sydney after 30 weeks of feeding with experimental diets. Blood was collected via cardiac puncture using EDTA-coated syringes and sample tubes, then centrifuged to isolate plasma. Aliquots of plasma were frozen on dry ice and stored at -80°C until analysis. The hearts and livers were obtained, weighed, and then clamped in liquid nitrogen temperature metal clamps, flash frozen and stored at -80°C until use. Cecum was obtained, weighed and flash frozen and stored at -80°C until use. Feces were collected prior sacrifice, freeze dried and stored at -80°C until use. One mouse was euthanized prior to the expected experimental endpoint due to the loss of body condition and was excluded from this study.

### Method details

#### Body composition

Longitudinal measures of body composition were assessed using the EchoMRI 900 (EchoMRI, TX, USA) after 28 weeks of feeding, prior to culling at each experimental endpoint.

#### Blood glucose and insulin measurement

Oral glucose tolerance tests (OGTT) were performed per cage of the mice two weeks before their sacrifice (i.e. 28 weeks after commencement of diets). Mice were subjected to 6 hours of morning fasting prior to testing. A clinical glucometer measured basal blood glucose through tail vein bleeding (Accu-Chek Performa, Roche Diagnostics Australia Pty Ltd). Fasted mice were administered glucose (2 g/kg lean mass) *via* oral gavage. Blood glucose was measured 15, 30, 45, 60 and 90 minutes after glucose administration. Blood insulin levels were determined by the Ultra Sensitive Mouse Insulin ELISA kit (Crystal Chem Inc). Homeostasis Model Assessment-Insulin Resistance (HOMA-IR) was used as an estimation for global IR for each group. It was calculated by multiplying the fasting plasma insulin (U/L) and fasting plasma glucose (mM) and divided by the constant.

#### Anesthesia for mice echocardiography

Echocardiography was performed in anaesthetized mice using 2-3% isoflurane in oxygen before imaging in the induction chamber and maintained anesthesia during imaging 1-2% isoflurane in oxygen delivered via a nose cone. During imaging, heart rate was maintained between 450-550 beats per minute (bpm), body temperature was kept at 36-37°C (via rectal probe). The mice were made to lie on their ventral side on a heated imaging platform. All animal studies were approved by the Institutional Animal Ethics Committee at the University of Sydney.

#### Echocardiography measurements

A Vevo2100 system high-resolution ultrasound system with a 40-MHz linear probe (FUJIFILM VisualSonics Inc., Canada) was used to perform echocardiography on mice at the end of 29 weeks of CHOW, HSD, HFD, and HFHSD. Several echocardiographic parameters including transmitral Doppler indices, Tissue Doppler imaging at the 4-chamber view, left atria area, and speckle training were used to assess diastolic function in mice. Echocardiographic measures of the diastolic function including mitral valve deceleration time (MVDT), peak early diastolic velocity (E), peak late diastolic velocity (A), peak early (E’) diastolic mitral annular velocity, *E/A* ratio, and isovolumetric relaxation time (IVRT) were recorded. Left ventricular wall thickness including interventricular septum wall thickness (IVST) and left ventricular posterior wall thickness (LVPWT), left ventricular end-diastolic dimension (LVEDd), left ventricular end-systolic diameter (LVESD) and left ventricular ejection fraction (LVEF) were also measured. All acquired images were digitally stored and assessed offline using the dedicated VisualSonics workstation.

#### Metabolite extraction from heart and liver tissues

Metabolite extraction from heart and liver tissues were achieved using a modified Bligh and Dyer method. Briefly, approximately 50 mg of crushed tissues were subjected to a three-phase extraction protocol involving tissue, ice-cold extraction medium (methanol:water, and chloroform). The aqueous layer was transferred into a new microfuge tube, concentrated in the Speed-Vac SPD120 (Thermo Fisher Scientific) and dried under nitrogen stream, followed by reconstitution in the acetonitrile/methanol/formic acid (75:25:0.2; v/v/v, HPLC grade; Thermo Fisher Scientific) for the HILIC analysis, and acetonitrile/methanol (25:25; v/v/v, HPLC grade) for the AMIDE analysis. All samples were stored at -80°C until further analysis by LC-MS/MS.

#### Metabolite extraction from plasma

Plasma samples were aliquoted for metabolomic analysis (10 uL HILIC, 30uL AMIDE), and samples were then stored at -80°C until use. Plasma samples were prepared by precipitating proteins with acetonitrile/methanol/formic acid (75:25:0.2, v/v/v, HPLC grade; Thermo Fisher Scientific) for the HILIC method, and acetonitrile/methanol (25:25; v/v/v, HPLC grade) for the AMIDE method. The extraction solvents also contained deuterated labelled internal standards. The HILIC extraction solvent contained 10 mM valine-d8 (98%; Sigma) and 25 mM phenylalanine-d8 (98%; Cambridge Isotope Laboratories, Inc), and the AMIDE extraction solvent contained 10 mM thymine-d4 (Sigma). Samples were vortexed and then centrifuged at 14 300 rpm at 4°C for 15 minutes to pellet proteins, and the metabolite-containing supernatant were transferred to HPLC-grade glass vials containing inserts (Waters).

#### Metabolite extraction from cecum and feces

Approximately 25 mg of mouse cecal and fecal samples underwent homogenization in a mixture of acetonitrile and water using a tissueLyser as part of the short-chain fatty acid (SCFA) extraction protocol. Following centrifugation, the supernatant was carefully collected and passed through a membrane filter (Nanosep 3K centrifugal device, Pall Corporation). All samples were stored at -80°C until further analysis using LC-MS/MS.

#### GT-derivatized SCFAs

Short chain fatty acids (SCFAs) including acetate, propionate, butyrate, valerate and caproate, underwent derivatization using Girard’s reagent T (GT) and 1-ethyl-3-(3-dimethylaminopropyl) carbodiimide (EDC). Each SCFA standard compound were initially dissolved in acetonitrile and subjected to derivatization with 100 mM GT and 100 mM EDC followed by an incubation period at 40°C. Samples were further diluted 20-fold in acetonitrile and analyzed in LC-MS/MS.

#### Targeted metabolomics analysis

For both HILIC and AMIDE analysis, LC-MS/MS system composed of an Agilent 1260 Infinity liquid chromatography (Santa Clara, CA, USA) system coupled to a QTRAP5500 mass spectrometer (AB Sciex, Foster City, CA, USA) was used. The polar metabolites in both positive and negative ionization mode were separated in hydrophilic interaction liquid chromatography (HILIC) mode using an Atlantis® HILIC column (Waters) and a XBridge™ AMIDE column (Waters), respectively, which allow the separation of metabolites of different properties. Sample analysis was conducted in a randomized sample order and data was acquired in the same batch on the same day.

The analysis software MultiQuant 3.0 (ABSciex) was used for multiple reaction monitoring Q1/Q3 peak integration of the raw data files (Analyst software, v.1.6.2.; ABSciex). The peak area corresponds to the abundance of that metabolite; the abundance values were then normalized to their bookended pooled tissue extracts in the subsequent analysis, which were included after every 10 study samples in the sample queue, to account for any temporal drift in instrument performance.

#### 16S microbiome

Total DNA was extracted from cecal samples using FastDNA™ SPIN Kit for Feces (MP Biomedicals Australasia) according to the manufacturer’s instructions. Briefly, cecal content was added to sodium phosphate buffer (825 μL) in a lysing matrix E tube (2mL), then topped up with PLS solution (275 μL). The mixture was homogenized (MP FastPrep™-24, 4m/s,15s), and centrifuged (14,000 x g, 5min) to remove the supernatant. After standard cleanup steps the DNA was collected in TES solution (60uL-100uL) and DNA concentration determined by a fluorometer (Qubit™ 2.0). A portion of stock DNA was diluted to 10ng/uL using Milli-Q water. Samples with a concentration less than 10ng/uL remained undiluted. Both the stock and diluted DNA solution were stored at -30^o^C until further use. 20uL of diluted DNA of each sample was sent to the Ramaciotti Centre for Genomics at University of New South Wales. The bacterial community was profiled using the 16S rRNA V4 region (515F/806R) by Illumina MiSeq.

Microbial analysis was performed in R v.4.2.2 using R studio. Sequence reads were aligned and partitioned into amplicon sequence variants (ASVs) using DADA2 v.1.24.0^47^ and default parameters. Then, paired-end sequence reads were aligned and partitioned into amplicon sequence variants (ASVs). Taxonomy was assigned to ASVs against the SILVA database v138.^48^ Multiple sequence alignment was performed with the msa package v.1.28.0,^49^ phylogenetic tree construction was performed with the phangorn package v.2.9.0^50^ and rooted using the ape package v.5.6-2.^51^

Microbial diversity analysis was performed with phyloseq package v.1.40.0^52^ and visualized with the ggplot2 package v.3.3.6.^53^ Prior to performing within-sample diversity (α-diversity), the microbial data was rarefied to even the sampling depth. α-diversity was measured with 3 different metrics (the inverse Simpson index, the Shannon index and the number of observed taxa) at 4 different taxonomy resolutions (Phylum, Family, Genus and ASV). Statistical analysis on α-diversity was performed with FSA package v.0.9.3 using Kruskal-Wallis test, and post-hoc test was done using Dunn’s test. Adjust p value was calculated using Benjamini-Hochberg Procedure.

Prior to comparing between-sample diversity (β-diversity), centred log-ratio (CLR) transformation was done with the microbiome package v.1.18.0.^54^ A pseudo count of 0.5 was applied across the dataset to allow for the CLR transformation.^55^ β-diversity was measured using the Bray-Curtis metric, weighted UniFrac metric and unweighted UniFrac using vegan package v.2.6-2.^56^ β-diversity distances between samples was visualized with principal coordinate analysis. Pairwise permutational multivariate analysis of variance (PERMANOVA) was performed using EcolUtils package v.0.1. The p-value was adjusted using Benjamini-Hochberg Procedure.

Differential abundance analysis was performed with ANOVA-Like Differential Expression (ALDEx) Analysis using ALDEx2 package v.1.28.1.^57^ Prior to performing ALDEx analysis, CLR transformation was done using the built-in function in ALDEx2 package. To find the significant bacteria taxa in desired groups, two selected datasets were compared using Kruskal Wallace test. The p-value was adjusted using Benjamini-Hochberg Procedure. The difference of bacterial taxa abundance was calculated using DESeq2 package v.1.36.0. Bacteria taxa were considered significant if the adjusted p-value was less than 0.05 and the log2 fold change was larger than 2 (i.e. more than 4 times larger). ALDEx2 result was visualized via either the built-in graphic tool in ALDEx2 package or via volcano plot using EnhancedVolcano package v.1.14.0.

To determine the feature-wise association of microbial ASVs and metabolomic phenotypes, multi-omic factor analysis v2 (MOFA+) was performed using MOFA2 package v.1.6.0.^58^^,^^59^ Prior to performing MOFA+ analysis, centred log-ratio (CLR) transformation was done on the microbial abundance counts with the microbiome package v.1.18.0^54^. A pseudo count of 0.5 was applied across the dataset to allow for the CLR transformation.^55^

MOFA+ uses Bayesian inference and probabilistic models to estimate latent factors and associated the feature weight matrices to explain the variance across dataset.^58^^,^^59^ MOFA+ results were visualized using the built-in tools in the MOFA2 package. The analysis was followed on the R codes available https://biofam.github.io/MOFA2.

### Quantification and statistical analyses

Data were analyzed in a two-by-two factorial framework, testing for main effects of high-fat, and high-sugar as well as a high-fat-high-sugar interaction, treating the chow-fed group as the control. Where we have repeated measures over time (e.g., body mass, food intake etc), outcomes were analyzed using a generalized additive mixed model (GAMM), treating animal ID and cage as random effects, time as a non-parametric smooth term, and dietary fat and sugar as parametric fixed effects with an interaction. For outcomes measured at a single time-point (i.e., without repeated measures), data were analyzed using linear-mixed effects models (LMMs) with cage as a random effect and dietary fat and sugar as fixed effects with an interaction. Data were analyzed and plots made in the statistical programming environment R, with GAMMs implemented using the ‘gam’ function in *mgcv*, and LMMs implemented using the ‘lmer’ function in *lme4*.^60^^,^^61^^,^^62^ To ascertain the statistical significance of main and interactive effects of the dietary exposures ANOVA-tables were created for models using the ‘anova’ function (*lmerTest* and *mgcv* packages). Data were visualized using *ggplot2*. Principle component analysis (PCA) was implemented using the ‘princomp’ function in R.
