# Supplementary material for: Multi-organ multi-omic and gut microbiome markers of fat and sucrose dietary oversupply in cardiometabolic disease
Source: iScience. 2025 Mar 1;28(4):111887. doi: 10.1016/j.isci.2025.111887 (PMC12131266; doi:10.1016/j.isci.2025.111887)
Supplement: Document S1. Tables S1 and S2 [file mmc1.pdf]

iScience, Volume *28*

Supplemental information

Multi-organ multi-omic and gut microbiome markers of fat and sucrose dietary oversupply in cardiometabolic disease

Ren Ping Liu, Alistair Senior, Zhen Bao, Yen Chin Koay, Andrew Holmes, and John F. O'Sullivan

Table S1: Composition of control (CHOW), high-sucrose (HS), high-fat (HF) and high-fat high-sucrose (HFS) diets. Related to Figure 1.

	**CHOW**	**HS**	**HF**	**HFS**
	**Diet composition by weight (g/100g)**			
**Ingredients**	**Per 100g as fed**	**Per 100g as fed**	**Per 100g as fed**	**Per 100g as fed**
Sucrose	10.000	48.901	7.047	21.697
Casein	20.000	20.000	25.289	25.296
Soya bean oil	7.000	7.000	6.997	6.999
Lard	-	-	15.143	15.148
Cellulose	5.000	6.900	3.099	3.799
Starch	40.441	9.690	28.687	17.098
Dextrinised starch	13.200	3.150	9.326	5.549
L Methionine	0.300	0.300	0.350	0.350
AIN_93_Trace Minerals	0.140	0.140	0.140	0.140
Fine Calcium Carbonate	1.313	1.313	1.311	1.312
Salt (Fine Sodium Chloride)	0.259	0.259	0.259	0.259
Potassium Dihydrogen Phosphate	0.686	0.686	0.690	0.690
Potassium Sulphate	0.163	0.163	0.163	0.163
Potassium citrate	0.258	0.248	0.250	0.250
AIN_93_Vitamins	1.000	1.000	1.000	1.000
Choline Chloride 75% w/w	0.250	0.250	0.250	0.250
**TOTAL**	**100.000**	**100.000**	**100.000**	**100.000**

Table S2: Components of Phenosignature Clusters. Related to Figure 7.

Cluster 1	Cluster 2	Cluster 3	Cluster 4
Liver_Thiamine	Plasma_Thiamine	Plasma_Acetylphosphate	Plasma_beta.Alanine
Fecal_Butyric.acid	Heart_Thiamine	Plasma_Hydroxyisocaproate	Cecum_Citrulline
Fecal_Propionic acid	Cecum_Thiamine	Plasma_Rhamnose	Cecum_Serotonin
Cecum_beta.Alanine	Fecal_Valeric.acid	Erysipelotrichaceae_Fecalibaculum_rodentium_asv2	Cecum_Aspartate
Heart_Homocysteate.HCA.	Cecum_Carnosine	Muribaculaceae_asv26	Cecum_Riboflavin
Heart_Oxaloacetate	Cecum_Phenylalanine	Muribaculaceae_asv14	Plasma_methyl.2.pyridone.5.carboxamide
Plasma_Aminoadipic.acid..AAD.	Cecum_Methionine	Muribaculaceae_asv17	Plasma_methyl.4.pyridone.5.carboxamide
Plasma_Riboflavin	Liver_Creatine	Lachnospiraceae_Lachnospiraceae NK4A136 group_asv153	Lachnospiraceae_asv13
Lachnospiraceae_Roseburia_asv105	Liver_trans.HYP	Lachnospiraceae_asv30	Lactobacillaceae_HT002_asv21
Clostridia vadinBB60 group_asv201	Lachnospiraceae_Lachnospiraceae NK4A136 group_asv51	Oscillospiraceae_Oscillibacter_asv86	Muribaculaceae_asv113
Lachnospiraceae_asv187	Bacteroidaceae_Bacteroides_thetaiotaomicron_asv35		Lachnospiraceae_Blautia_coccoides_asv46
Desulfovibrionaceae_Desulfovibrio_asv10	Lachnospiraceae_GCA-900066575_asv160		Lachnospiraceae_asv25
Lachnospiraceae_Lachnospiraceae NK4A136 group_asv42	Muribaculaceae_asv7		Bifidobacteriaceae_Bifidobacterium_pseudolongum_asv3
Lachnospiraceae_Lachnoclostridium_asv64	Muribaculaceae_asv20		Lachnospiraceae_Roseburia_asv219

Lachnospiraceae_Lachnospiraceae NK4A136 group_asv99	Lachnospiraceae_asv125		Lachnospiraceae_asv15
Ruminococcaceae_asv140	Lachnospiraceae_GCA-900066575_asv72		Lactobacillaceae_Lactobacillus_johnsonii_asv6
Lachnospiraceae_asv121	Lachnospiraceae_Lachnoclostridium_asv148		Ruminococcaceae_Incertae Sedis_asv91
Lachnospiraceae_Roseburia_asv155			Atopobiaceae_Coriobacteriaceae UCG-002_asv5
Lachnospiraceae_asv178			Lachnospiraceae_asv159
Clostridia UCG-014_asv233			Lachnospiraceae_asv11
Lachnospiraceae_asv90			Lachnospiraceae_Lachnospiraceae FCS020 group_asv146
Oscillospiraceae_asv22			
Oscillospiraceae_Colidextribacter_asv56			
Lachnospiraceae_Tuzzerella_asv67			
Muribaculaceae_asv68			
Oscillospiraceae_asv194			
Lachnospiraceae_Lachnospiraceae NK4A136 group_asv126			
Lachnospiraceae_asv70			
Lachnospiraceae_Lachnoclostridium_asv144			
Lachnospiraceae_Roseburia_asv83			
Lachnospiraceae_Roseburia_asv34			
Lachnospiraceae_[Eubacterium]			

xylanophilum group_asv204			
Peptostreptococcaceae_Romboutsia_ilealis_asv78			
Peptostreptococcaceae_Romboutsia_ilealis_asv62			
Lachnospiraceae_asv136			
